# The study on the morphological changes of oropharynx in patients with complete unilateral cleft lip and palate after palatopharyngeal closure

**DOI:** 10.3389/fnins.2022.997057

**Published:** 2022-09-29

**Authors:** Baitong Chen, Hongchuang Zhang

**Affiliations:** Xuzhou First People's Hospital, Xuzhou, China

**Keywords:** velopharyngeal closure, oropharyngeal morphology, airway volume, cleft lip and palate, palatopharyngeal closure

## Abstract

Cleft lip and palate can be treated as one of the most common craniofacial congenital malformations in humans. Such disease influences tens of millions of patients all over the world. Cleft lip and palate deformity affects many important physiological functions, including breathing, swallowing, speech, chewing, and aesthetics. This work focuses on investigating the morphology and airway volume of oropharynx patients with unilateral complete cleft lip and palate after palatopharyngeal closure. In addition, this work evaluated the similarities and differences between patients with cleft lip and palate and those without such an issue. The employed data, selected from the Department of Stomatology of Xuzhou First People's Hospital, are based on the conical beam CT images. The study sample was divided into two groups: the selected experimental group, who confronted the cleft lip, cleft palate, and velopharyngeal closure surgery, and the selected control group, who are healthy children at the corresponding age. The parameters, including the airway volume, the airway volume of velopharyngeal and oropharyngeal segments, the minimum cross-sectional area of the pharynx, the horizontal plane airway area of the hard palate and soft one, the horizontal airway area of the hyoid bone, and the vertical distance between the hard palate and soft palate, can be measured by Dolphin. These parameters were analyzed with a statistical approach. The analysis of the above-mentioned parameters reveals that the airway volume, the minimum cross-sectional area of the pharynx, the horizontal cross-sectional area of the hyoid, and the distance between the hard palate and soft palate tip in patients with complete unilateral cleft lip and palate show significant differences between the experimental group and the control group. Meanwhile, other parameters, including the horizontal cross-sectional area of the airway in the horizontal plane of the hard palate and the horizontal plane of the soft palate, did not show noticeable differences in the two groups. The patients, who confronted the unilateral complete cleft lip and palate, can improve with the velopharyngeal closure surgery. Furthermore, the length and vertical distance of the soft palate and the volume of each segment of the airway exhibit differences between the experimental group and the control group.

## Introduction

Cleft lip and palate (CLP) is the most common congenital maxillofacial deformity, whose morbidity rate can reach more than 0.1% (Mossey et al., 2009; Akarsu-Guven et al., 2019; Yatabe-Ioshida et al., 2019). These diseases can be classified into two subtypes: unilateral cleft lip (UCL) and bilateral cleft lip and palate (BCLP) (Vieira, 2008). It is noted that the cleft palate is the common type. According to the latest reports, the percentage of unilateral complete cleft lip and palate is much higher than the bilateral ones (Dixon et al., 2011). Therefore, it is urgent that the cleft lip and palate needs sequential treatment during early childhood (Roy et al., 2019; Yilmaz et al., 2019). However, such treatment includes several procedures, such as preoperative orthodontic treatment, surgical treatment, speech therapy, velopharyngeal closure surgery, postoperative orthodontic treatment, and other related ones (Pai et al., 2019). Nevertheless, these treatments are associated with some shortcomings, including postoperative scar contracture and abnormal muscle strength with the operation. What is worse is that the majority of these patients confront abnormal dentition development and insufficient jaw development, which have negative influences on the teeth, jaw, and face. The patients' fractured side bone tissue defects may lead to soft palate shortening, postoperative soft tissue scar formation, and several abnormal symptoms, such as insufficient maxillary development, posterior mandibular rotation, deviated nasal septum, velopharyngeal insufficiency, upper airway deformity, mouth breathing, insufficient breathing during sleep, increased snoring, and abnormal tongue position. These patients often confronted snoring and dyspnea during their sleep (Yates et al., 2020). According to the Sarmadi's efforts, the adenoids levels of adolescent patients with UCL can be far higher than others of the same age (Salgado et al., 2019). Meanwhile, it is noted that the risk of several diseases in these patients, including cardiovascular, cerebrovascular diseases, and hypertension, is much higher than observed in healthy individuals. Even more serious condition is that these patients show obvious daytime sleepiness. Moreover, the CLP has a dangerous influence on the function of the pharyngeal airway (Bittermann et al., 2021).

The uraniscolalia has a negative influence on patients' quality of life. The majority of uraniscolalia cases are mainly caused by velopharyngeal incompetence (VPI) (Rosenthal et al., 2018). Speech therapy is a significant element in the treatment of CLP. Nevertheless, there are still several velopharyngeal insufficiency patients with cleft palate repair. Therefore, the closing rate of the palatal pharynx can be employed as an effective measurement for further treatment. More than 85% of CLP patients confront the dangerous signs of dysarthria. Therefore, some patients encounter the communication obstacle as the asaphia. According to the suggestions of the American Cranio-Maxillofacial Association (ACPA), the speech and velopharyngeal function can be treated as the evaluation index before CLP operation (Al-Namankany and Alhubaishi, 2018; Kapadia et al., 2020). Therefore, the velopharyngeal closure rate can be employed to evaluate the effect of the second-stage operation. Upper airway stenosis may have negative influences on the functions of breathing and ventilation. Such a situation may lead to a change in the breathing forms, such as mouth breathing. It is pointed out that long-term mouth breathing may lead to the deformity of the jaw and dentition, which is the clockwise rotation of the mandible and hyoid in different degrees. It also contributes to the vertical development of the maxillofacial region. Considering the interactions between airway morphology and craniofacial morphology, abnormal craniofacial bone morphology may affect the airway structure during childhood. Therefore, the dentition CLP patients' upper airway morphologies can more easily distinguish than other patients' CLP upper airway morphologies.

Furlow's double-reverse Z-operation demonstrated two functions, including the soft palate through Z-shape and prevents longitudinal scar contracture (Kara et al., 2021). Posterior pharyngeal flap transfer repair has been widely utilized in the treatment of velopharyngeal insufficiency (Al-Fahdawi et al., 2018; Aslan et al., 2018; Worley et al., 2018; Hardin-Jones et al., 2020; Hi'ilani et al., 2020; Kim et al., 2022), and is considered to be one of the most effective treatments to deal with velopharyngeal insufficiency (Rose et al., 2002; Mink van der Molen AB, 2009; Reiser et al., 2011; Ji et al., 2019; Zaluzec et al., 2019; Zhao et al., 2021). In this work, we focus on evaluating the efficacy of Furlow's double-reverse Z-operation combined with posterior pharyngoplasty in the treatment of velopharyngeal insufficiency after cleft palate surgery. Using this method, we aim at the reconstruction of palatine levator muscle, prolong soft palate, and narrow pharyngeal cavity. In this work, CBCT can be employed to collect the velopharyngeal images of patients after cleft palate surgery. Three-dimensional reconstruction can be employed to study the velopharyngeal closure of patients, which is more accurate than the cephalometric lateral films. It can reflect the morphology and dynamics of velopharyngeal closure in patients with three-dimensional reconstruction, and provides a more intuitive reference for clinicians to choose appropriate surgical methods. Moreover, CBCT can be utilized to observe pharyngeal morphology. Compared with the traditional nasopharynx fibroscope and speech pronunciation, this method was simpler, direct, and painless, and showed high detection accuracy. This study mainly compared the volume and morphology of the oropharyngeal area in patients with CLP after velopharyngeal closure and those without CLP. This work analyzed the airway abnormalities and possible causes in patients with cleft lip and palate.

## Materials and methods

**Figure 1:**
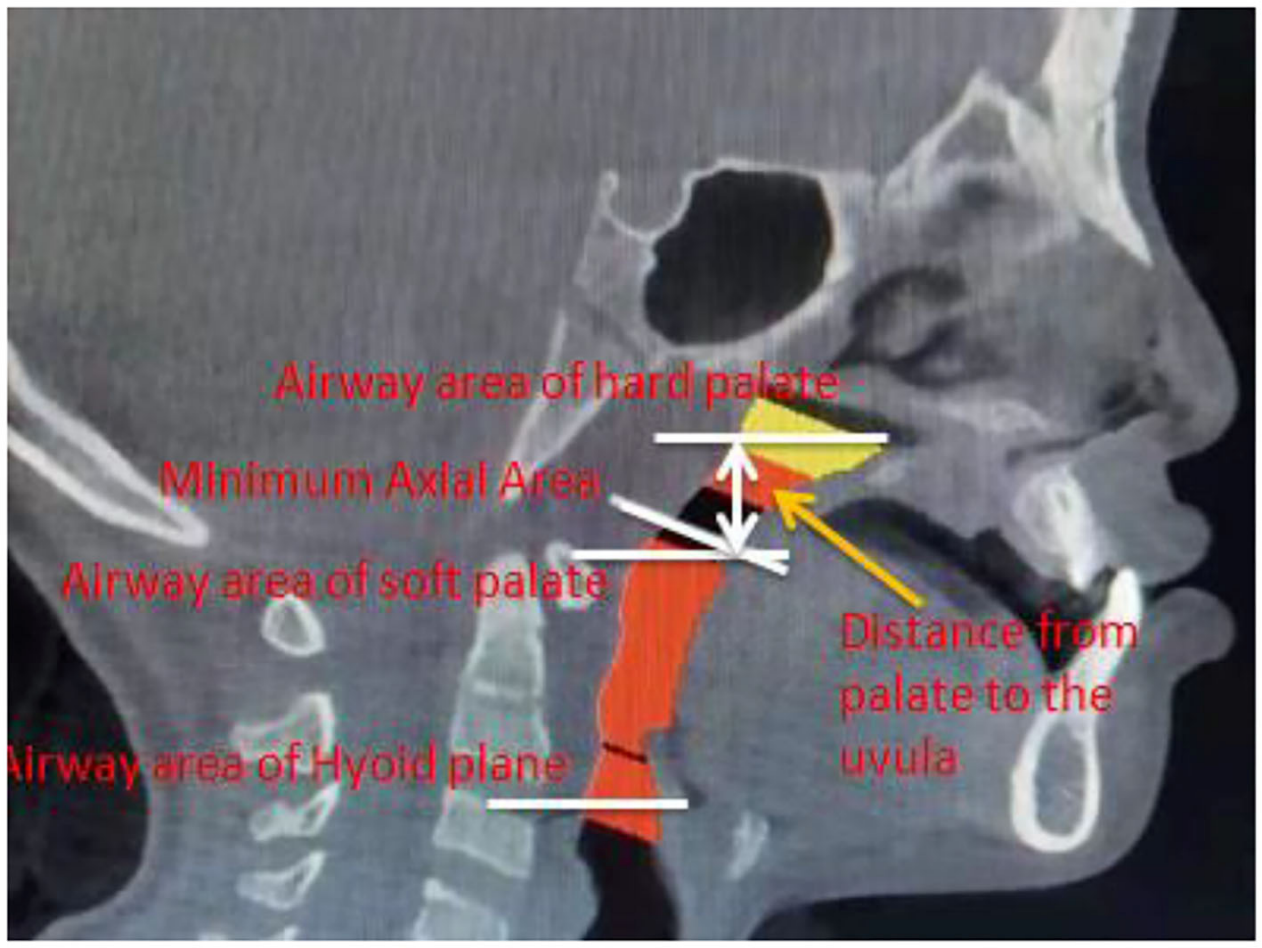
Airway volume and measurement plane of the oropharynx.

**Figure 2:**
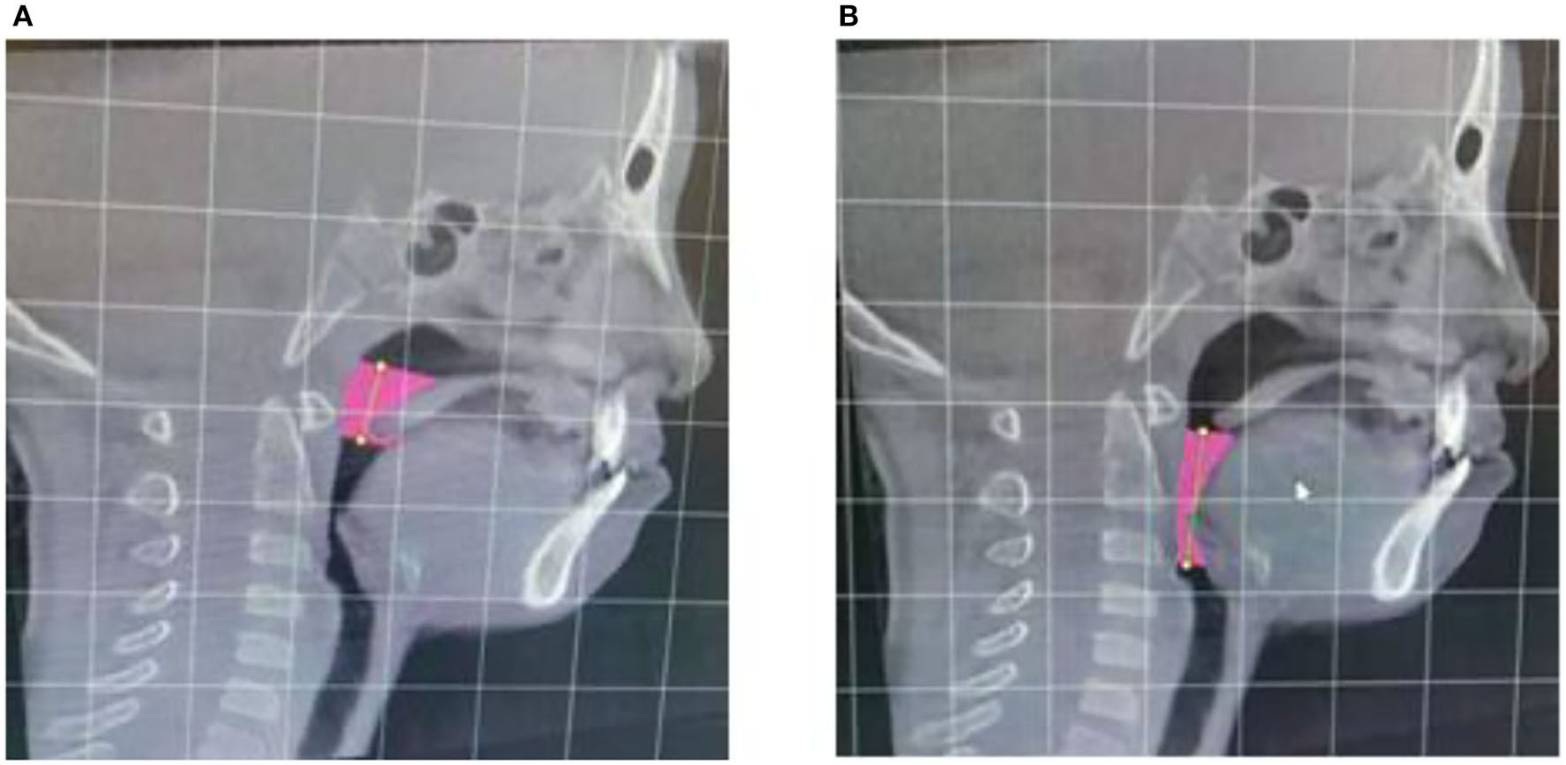
Airway volume of palatopharyngeal segment **(A)** and airway volume of glossopharyngeal segment **(B)**.

**Figure 3:**
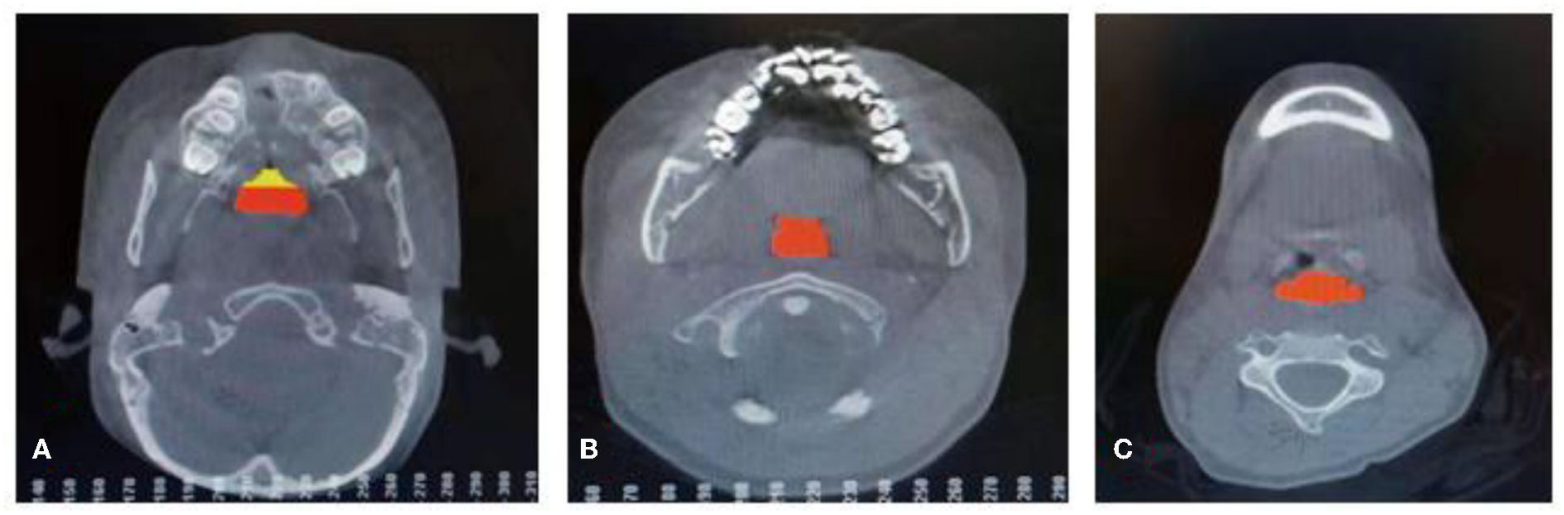
Airway area at the level of the hard palate **(A)**, airway area at the level of the soft palate **(B)**, and airway area at the level of the hyoid bone **(C)**.

The CBCT approach has been employed to scan all the analyzed patients in the Department of Stomatology, Xuzhou First People's Hospital. When it comes to cone beam CT (CBCT), this kind of CT is the most promising and practical equipment in oral skull imaging equipment. This approach, which shows a high spatial resolution, has been widely utilized in the field of stomatology. The amount of radiation released by CBCT is very low, which is only equivalent to 1/30–1/40 of the traditional CT. CBCT has been extensively used in planting, teeth, orthodontics, orthognathic, periodontal, and other related issues. During the CBCT, all the patients should sit up straight. Their orbital ear planes should be parallel to the ground. The upper and lower dentitions of patients should be bitten in the median bite position. Moreover, these patients should keep quiet breathing without swallowing. All patients have been photographed using the same procedures and parameters. The whole CT images can be analyzed by Dolphin Imaging software, and the mark points were determined. The same doctors completed all the above-mentioned procedures. The measurement issues, including the airway volume and measurement plane of the oropharynx, airway volume segments and airway area at different levels, are demonstrated in Figures 1–3.

**Figure 4:**
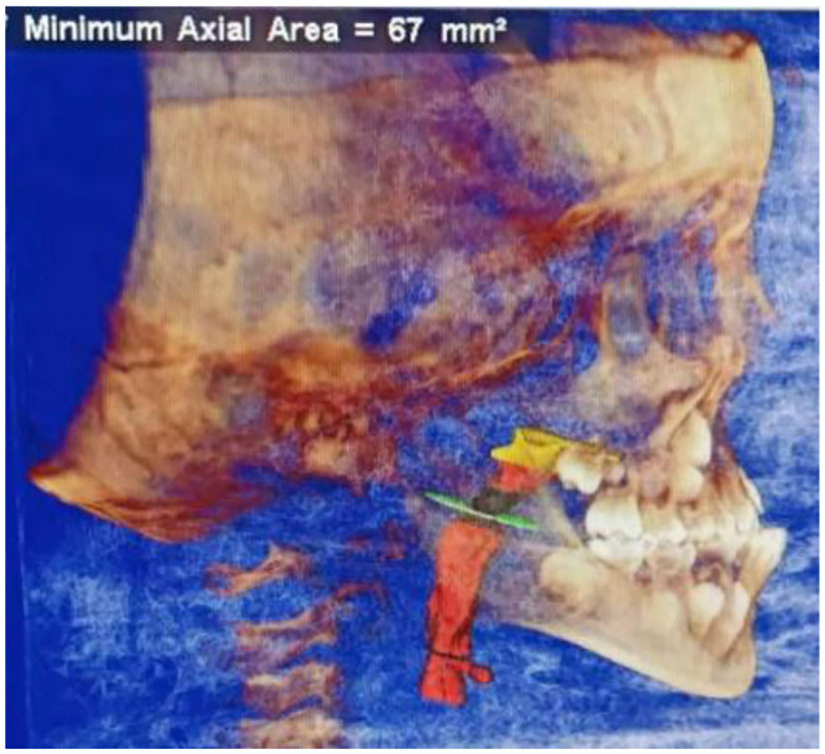
Three-dimensional reconstruction of airway.

During this work, the main measurement issues, including airway volume, airway volume of the palatopharyngeal segment, airway volume of the glossopharyngeal segment, minimum airway area, horizontal airway area of the hard palate, horizontal airway area of soft palate tip, horizontal airway area of the hyoid bone, and distance between the hard palate and soft palate tip, can be demonstrated. Considering these issues, the reconstruction of the airway is shown in Figure 4.

## Results

**Figure 5:**
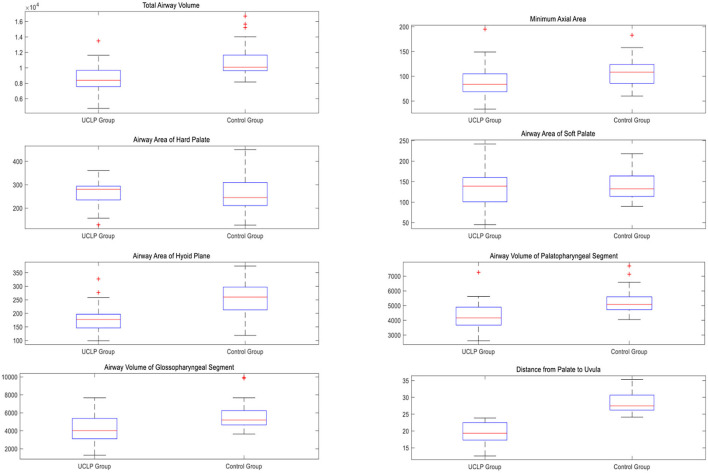
The dependent variables in the unilateral complete cleft lip and palate group and control group.

Hypothesis testing is an important concept in the field of statistics. Such issue is an important evidence to determine the original hypothesis is correct. The *p*-value can be evaluated by the method of a significance test. When the *p*-value is lower than 0.05, the difference in error probability between the two groups is < 0.05. In this work, the SPSS 20.0 statistical software was employed to analyze the data of these patients (O'connor, 2000). The measurement data can be expressed as mean ± standard deviation (Chang et al., 2017; Bao et al., 2020, 2022). The one-factor analysis of variance was utilized to compare the two groups. When the *p*-value was lower than 0.05, the difference was statistically significant (Sobral et al., 2018). In this work, CBCT was utilized to photograph all samples. A total of 100 samples were divided into two groups: the experimental group and the control group. The experimental group included 50 patients with unilateral complete cleft lip and palate after velopharyngeal closure, and the control group included 50 patients with non-left lip and palate. The age of these 100 patients ranged from 9 to 11 years, and the gender ratio of the experimental group was similar to that of the control group. The *p*-value of each group was higher than 0.05, which means their variances are homogeneous. All the samples were analyzed with the variance analysis approach. The results show that changes in the airway volume were statistically significant (*p_value* < 0.05) between the experimental group and the control group. The control group's measurements, including total airway volume, airway volume of the palatopharyngeal segment, and airway volume of the glossopharyngeal segment, were higher than the control ones. In addition, the control group's measurements, including the cross-sectional area, the minimum cross-sectional area of the airway, and the airway area at the tongue level, were significantly larger than in the experimental group. It was noted that there was no significant difference in the horizontal cross-sectional area of the hard palate and the horizontal cross-sectional area of the soft palate between the experimental and control groups. Therefore, the measurements, including the distance between the hard palate and the soft palate, are statistically significant between the two groups. The detailed information is presented in Table 1, and the comparison between the unilateral complete cleft lip and palate group and the control group is shown in Figure 5.

**Table 1:** Oropharyngeal airway measurement indexes of unilateral complete cleft lip and palate group and control group.

**Dependent variables**	***F***	***P_value***
Total airway Volume	28.785	0.001 < *
Airway volume of palatopharyngeal segment	24.84	0.001 < *
Airway volume of glossopharyngeal segment	18.926	0.001 < *
Minimum Axial Area	10.229	0.002*
Airway area of hard palate	0.054	0.817
Airway area of soft palate	0.044	0.834
Airway area of Hyoid plane	27.543	0.001 < *
Distance from palate to uvula	187.58	0.001 < *

*Means the *p*-value is lower than 0.05.

From Figure 5, we can observe that the value of total airway volume ranges from 4,370 to 13,490 in the unilateral complete cleft lip and palate group, while the value ranges from 8,163 to 16,696 in the control group. The minimum axial area values range from 27 to 195 in the test group and from 60 to 183 in the control group. The airway area of hard palate values ranges from 129 to 361 in the test group and from 128 to 450 in the control group. The airway area of soft palate values ranges from 45 to 242 in the test group and from 90 to 218 in the control group. The airway area of the hyoid plane ranges from 99 to 327 in the experimental group and from 119 to 375 in the control group. The airway volume of the palatopharyngeal segment ranges from 2,615 to 7,257 in the test group and from 4,054 to 7,692 in the control group. The airway volume of the glossopharyngeal segment ranges from 1,281 to 7,677 in the test group and from 3,642 to 9,934 in the control group. The distance from the palate to the uvula ranges from 12.6 to 23.9 in the test group and from 24.1 to 35.3 in the control group.

## Discussion

Velopharyngeal closure means that when pronouncing non-nasal consonants, the soft palate is in contact with the posterior wall of the pharynx, separating the oropharynx from the nasopharynx, so as to facilitate normal pronunciation (Silvestre et al., 2014). When velopharyngeal insufficiency occurs, the nasal cavity and oral cavity cannot be completely separated, which affects people's normal pronunciation and speech (Zimmerman et al., 2019; Apriani et al., 2020; Gurgel et al., 2022). In order to change this situation, patients with velopharyngeal insufficiency underwent velopharyngeal closure surgery to extend the soft palate and narrow the pharyngeal cavity, which can help them to achieve good velopharyngeal closure (Abdelkarim et al., 2021). However, there is no literature regarding whether or not this operation has a greater impact on the development of glossopharyngeal soft and hard tissues and airway volume in children. Obstructive sleep apnea-hypopnea syndrome (OSAHS) is the result of the obstruction of the upper respiratory tract due to structural and functional abnormalities during sleep. The incidence of OSAHS can range from 4 to 7.3% in China (Astani et al., 2018). Although obesity is considered to be the major risk factor for OSAHS, upper airway and craniofacial morphology are also important factors in the pathogenesis of OSAHS. For example, abnormal development of the mandible or maxilla suggests that the bone may limit airway patency.

The results showed that the total airway volume, velopharyngeal airway volume, and glossopharyngeal airway volume in the unilateral complete cleft lip and palate group were significantly different from those in the non-cleft lip and palate group. The velopharyngeal airway volume and glossopharyngeal airway volume in the unilateral complete cleft lip and palate group were significantly smaller than those in the non-cleft lip and palate group. Therefore, patients with cleft lip and palate were more likely to have sleep apnea syndrome, which was consistent with the results of Sobral DS and Silvestre J et al. (Ertaş and Ataol, 2019).

With regard to the morphology and volume of the velopharyngeal airway, we found no significant difference between the cleft lip and palate group and the non-cleft lip and palate group by measuring the airway area at the horizontal plane of the hard palate and the soft palate tip. This result suggests that Furlow's double-reverse Z-operation + posterior pharyngeal flap angioplasty has a good surgical effect on improving the velopharyngeal morphology of patients by increasing the thickness of the soft palate caused by scar contracture. Furlow's double-reverse Z-operation lengthens the soft palate while preventing scar contracture of longitudinal soft tissue. Sommerlad palatal levator muscle reconstruction rearranges from the abnormal longitudinal anatomic position to a transverse position by changing the position of the palatal levator muscle, so as to avoid maxillary growth and development deformity caused by scar formation and effectively prolong the length of the soft palate.

However, when measuring the vertical distance, we found that the vertical distance from the hard palate to the soft palate tip in patients with cleft lip and palate was significantly shorter than that in patients without cleft lip and palate. We speculate that the possible reasons were as follows: (1) the extension of the soft palate is still insufficient, and the distance from the hard palate to the tip of the soft palate is relatively short; (2) insufficient maxillary length and vertical growth deficiency due to the bone defects. Therefore, we expect to find an operation that can better extend the length of the soft palate, and increased orthodontic treatment in the growth and development of children can better promote the development of maxillary anterior and posterior, and vertical teeth.

By measuring the shape and volume of the oropharyngeal airway, we found that the cross-sectional area of the horizontal airway in the cleft lip and palate group was significantly smaller than that in the control group, and the oropharyngeal volume was also significantly reduced, which was similar to previous efforts (Mehta et al., 2018; Ertaş and Ataol, 2019). The reason for this phenomenon may be that the hyoid bone is displaced backward in patients with cleft lip and palate, and the airway cross-sectional volume at the level of the hyoid bone decreases. The change in the position of the hyoid bone is an important cause of the onset of obstructive sleep apnea syndrome. The change in the position of the hyoid bone leads to changes in the position and shape of the tongue muscles, thereby affecting the area of the pharyngeal airway. Studies have shown that the tilt angle of the tongue and the position of the bone may be related to the position of the mandible (Vakili et al., 2022). We will further study the vertical and tilt angles of the hyoid bone after velopharyngeal closure, and explore the relationship between the hyoid muscle, hyoid bone, mandible, and airway. It provides a more meaningful reference value for the further selection of tongue muscle training methods and orthodontic treatment programs for children with cleft lip and palate.

## Conclusion

Complete cleft lip and palate can be regarded as the most common congenital maxillofacial deformity. This work focus on investigating the morphology and airway volume of oropharynx patients with unilateral complete cleft lip and palate after palatopharyngeal closure. In addition, this work evaluated the similarities and differences between patients with cleft lip and palate and those without such an issue. This study was a retrospective study. The employed data, selected from the Department of Stomatology of Xuzhou First People's Hospital, are based on the CBCT images. The study sample included two groups: the selected experimental group, who confronted the cleft lip, cleft palate, and velopharyngeal closure surgery, and the selected control group, who are healthy children at the corresponding age. The parameters, including the airway volume, the airway volume of velopharyngeal and oropharyngeal segments, the minimum cross-sectional area of the pharynx, the horizontal plane airway area of the hard palate and soft one, the horizontal airway area of the hyoid bone, and the vertical distance between the hard palate and soft one, can be measured by using Dolphin software. Then, these parameters were analyzed using a statistical approach.

In total, Furlow's double-reverse Z-operation + posterior pharyngeal valvuloplasty can better improve the velopharyngeal morphology and reduce the increase in soft palate thickness caused by scar contracture, among patients with unilateral complete cleft lip and palate. However, the extension of the soft palate still needs further improvement. The position of the hyoid bone in patients with cleft lip and palate moves back, and the volume of the oropharyngeal airway is reduced. Therefore, we should intervene in orthodontic treatment and tongue muscle training in the early stage to improve the airway. Secondary facial deformities and dysfunctions caused by insufficient volume enable patients with cleft lip and palate to obtain better facial appearance and oral function. Furlow's double-reverse Z-operation+ posterior pharyngeal flap surgery can improve the velopharyngeal morphology of patients with cleft lip and palate. However, there are still shortcomings in the extension of the soft palate. Patients with cleft lip and palate should receive tongue muscle training as soon as possible to improve tongue position and obtain better airway ventilation. Therefore, several efforts should be proposed in this future work.

## Data availability statement

The original contributions presented in the study are included in the article/supplementary material, further inquiries can be directed to the corresponding author.

## Author contributions

BC analyzed the data of patients. HZ collected the data of these patients. All authors edited the manuscript.

## Funding

This work was supported by the Fundamental Research Funds for the Central Universities (2020QN89) and Xuzhou Science and Technology Plan Project (KC19142 and KC20117).

## Conflict of interest

The authors declare that the research was conducted in the absence of any commercial or financial relationships that could be construed as a potential conflict of interest.

## Publisher's note

All claims expressed in this article are solely those of the authors and do not necessarily represent those of their affiliated organizations, or those of the publisher, the editors and the reviewers. Any product that may be evaluated in this article, or claim that may be made by its manufacturer, is not guaranteed or endorsed by the publisher.
